# Case report: Application of non-VKA oral anticoagulants in patient of idiopathic hypereosinophilic syndrome with intracardiac thrombus

**DOI:** 10.3389/fphar.2022.1018394

**Published:** 2022-09-19

**Authors:** Man Zou, Geyan Liu, Yanhui Li

**Affiliations:** ^1^ Department of Oncology, Tongji Hospital, Tongji Medical College, Huazhong University of Science and Technology, Wuhan, China; ^2^ Department of Internal Medicine, Tongji Hospital, Tongji Medical College, Huazhong University of Science and Technology, Wuhan, China

**Keywords:** idiopathic hypereosinophilic syndrome, non-VKA oral anticoagulants, shortness of breath, heart failure, intracardiac thrombus, case report

## Abstract

Idiopathic hypereosinophilic syndrome (IHES) is a rare but life-threatening disease related to a group of myeloproliferative disorders characterized by prolonged eosinophilia of unknown cause and inflammatory damage to multiple organs. Here, we present a 44-year-old female patient complaining of shortness of breath and palpitations for 1 month. Her history and presentation were unremarkable, except for a 3-years history of rheumatoid arthritis treated with ibuprofen (0.3 g per day). Initial examination showed heart rate (HR) 120 bpm, respiratory rate (RR) 20 bpm, temperature (T) 36°C, blood pressure (BP) 130/70 mmHg, ventricular gallop rhythm, rales at the lung bases, soft abdomen, nonpalpable liver and spleen, and slight edema in both lower extremities. Bone marrow aspirate and biopsy confirmed the diagnosis of IHES, while cardiac MRI showed intracardiac thrombus. The symptoms of shortness of breath and palpitation disappeared, the eosinophil counts in routine blood tests were normal, and the thrombus in the cardiac cavity gradually disappeared after combined therapy of anti-hypereosinophilic, anti-coagulant and anti-heart failure treatments.

## Introduction

Idiopathic hypereosinophilic syndrome (IHES) is a myeloproliferative disorder characterized by persistent eosinophilia. The occurrence of this disease is rare, but it often causes serious damage to multiple organs and has high mortality (He et al., 2020; Abo Shdid et al., 2021; Wang et al., 2021). It was initially documented by Anderson and Hardy in 1968 (Anderson and Hardy, 1968), while the diagnosis of IHES was first defined by Chusid et al. (1975). The diagnosis of this disease usually includes the following criteria (Abo Shdid et al., 2021): 1) the peripheral eosinophil count is more than 1.5 × 10^9/L for more than 6 months; 2) other diseases leading to increased eosinophils, such as parasitic diseases, allergic diseases, drug allergies, lymphoma, HIV infection, and nonhematologic malignancies, are excluded; and 3) there are clear symptoms and signs of organ dysfunction due to eosinophil infiltration, such as hepatosplenomegaly, heart failure, heart murmur, diffuse and local neurological sign abnormalities, pulmonary fibrosis, fever, weight loss and anemia. Multiorgan involvement is frequently noted in these patients.

It has been reported that eosinophil-mediated cardiac involvement occurs in approximately 60% of IHES patients and is the leading cause of related deaths (Mansour et al., 2018; He et al., 2020). Heart failure is usually reported in IHES patients; however, IHES accompanied by intracardiac thrombus and treatment with new oral anticoagulants (NOACs) is less common. Here, we report a case of IHES with heart failure and cardiac apex thrombus treated with rivaroxaban, aiming to improve the treatment and prognosis of this rare but life-threatening disease.

## Case presentation

Medical history summary: A 44-year-old woman was hospitalized for “shortness of breath and palpitation for 1 month.” She had been in her usual state of health until 1 month earlier when she had exertional dyspnea and palpitations. Her history and presentation were unremarkable, except for a 3-years history of rheumatoid arthritis treated with ibuprofen 0.3 g daily. Her initial temperature was 36.5°C, pulse was 120 bpm, respiratory rate was 20 bpm, blood pressure was 130/70 mmHg, and oxygen saturation in room air was 96%. Physical examination showed ventricular gallop rhythm, rales at the lung bases, soft abdomen, nonpalpable liver and spleen and slight edema in both lower extremities. Routine blood tests showed normal WBC and RBC counts (6.77 × 10^^9^/L and 3.99 × 10^^9^/L) with increased eosinophils (2.06 × 10^^9^/L↑), normal hemoglobin (Hb 121 g/L) and decreased platelet counts (PLT 62 × 10^^9^/L↓). Other abnormal lab results included anti-CCP ↑, RF ↑, PANCA (+), BNP 4000 pg/ml ↑ and cTnI 0.03 mg/L ↑. We also conducted tests for urine, stool, liver function, kidney function, ESR, CRP, IL-5, anti-phospholipid antibodies, thyroid function, and tumor markers, the results of which were all normal.

**FIGURE 1:**
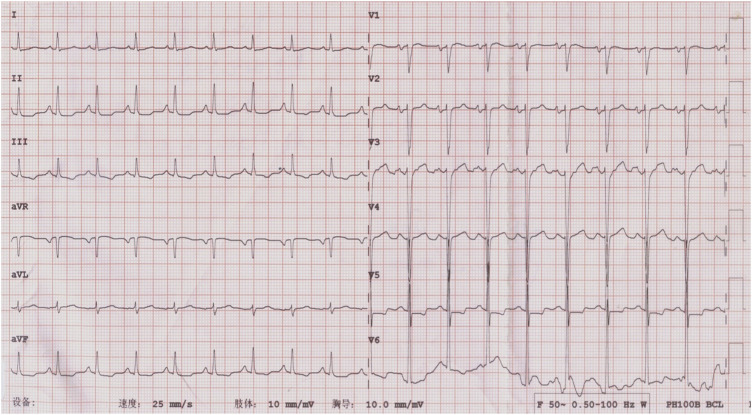
ECG at admission, showing flat T wave in I, II, III, AVF, and V1 to V6; ST segment depression in II, III, AVF, and V5; and poor R wave progression in the thoracic leads (V1-V4).

**FIGURE 2:**
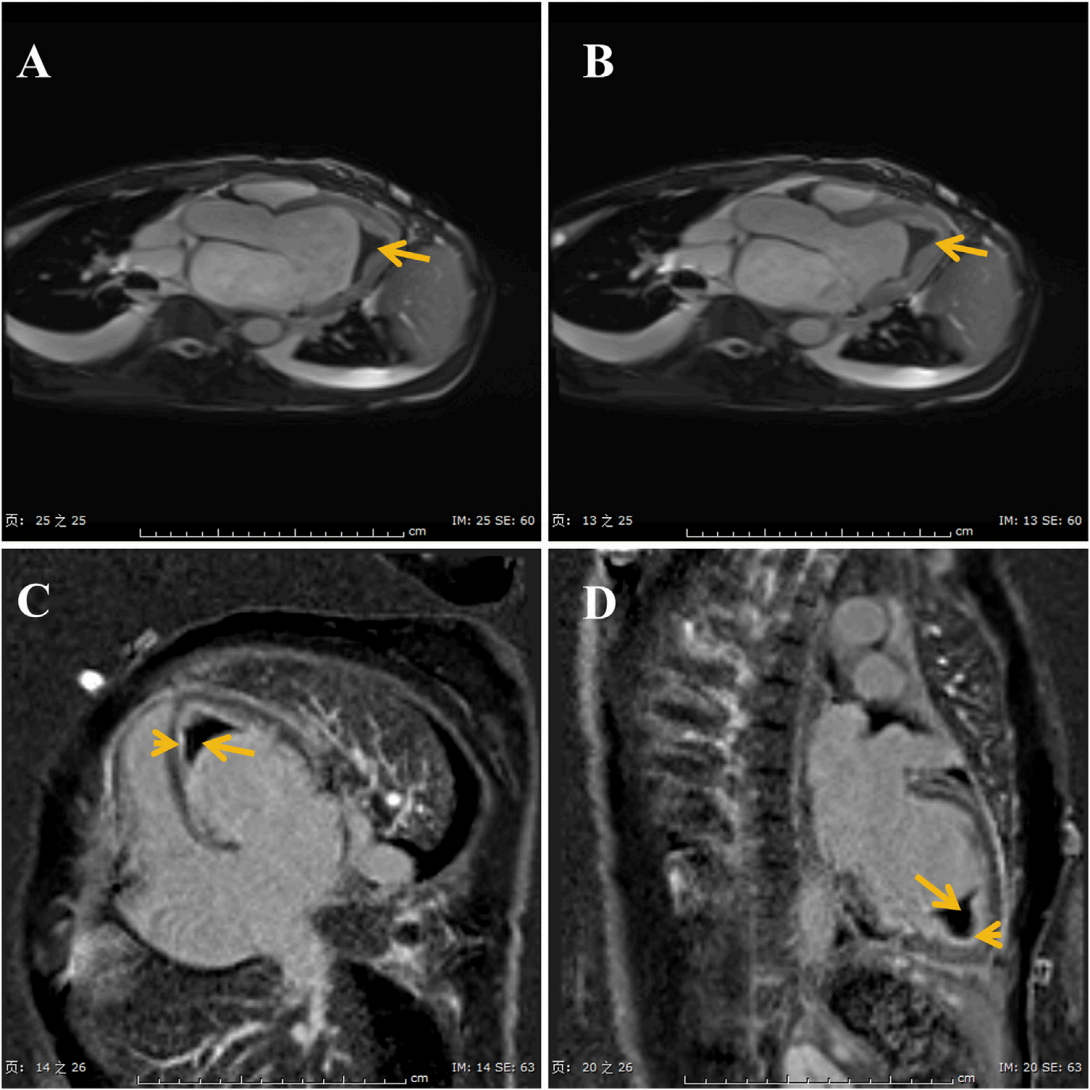
Images of cine-cardiac magnetic resonance show the thrombi (long arrows) in the apex **(A,B)**. Gadolinium enhanced magnetic resonance imaging show late gadolinium enhancement is in the endocardial layer **(C,D)**.

**FIGURE 3:**
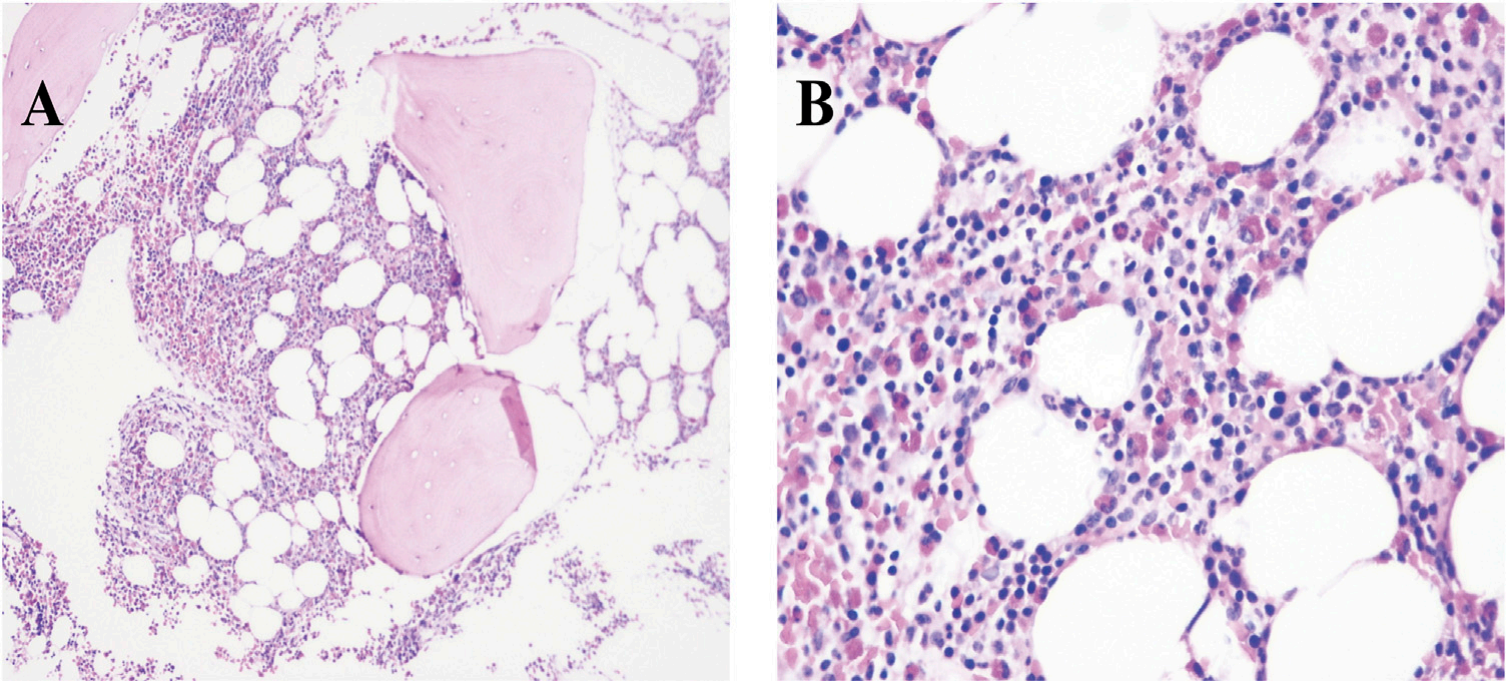
Photomicrograph with different magnifications (**A**, ×10 and **B**, 40×) of bone marrow biopsy showing significant proliferation of eosinophilia (deep pink cells).

ECG showed sinus tachycardia, ST-T variation, and poor R wave progression in chest leads (Figure 1). Portable 24-h ECG Holter revealed ischemic ST-T changes but no severe cardiac arrythmia. Echocardiography revealed an abnormal endocardial surface of the left and right ventricles (further examination is recommended), mild to moderate mitral regurgitation and pericardial effusion. Thickness measurements were normal, as was left ventricular systolic function (EF 54%). Chest CT revealed a dilated left lower bronchus and localized emphysema in the right lower lung, but the lung function evaluation for this patient was normal. Further cardiac MRI tests reported subendocardial myocardial infarction (MI) in the left ventricular free wall, thrombosis in the left and right ventricular apex, mitral regurgitation, left atrial enlargement and pericardial effusion (Figure 2). To confirm whether the patient had MI or pulmonary embolism (PE), we performed coronary artery angiography and pulmonary CT angiography (CTA). However, there was only mild atherosclerosis in the left anterior descending artery and no embolus in pulmonary artery. In addition, bone marrow aspirate showed a significantly increased amount of eosinophil cells (16.5%) but without bone marrow fibrosis. Bone marrow biopsy indicated that hematopoietic cells were proliferating and active, mainly eosinophils (Figure 3). We further performed flow cytometry and found no leukemia fusion gene. Final serology for parasites was also unrevealing.

Our final diagnosis was 1) idiopathic eosinophilia (moderate); 2) intracardiac thrombus; and 3) HFpEF. This patient was administered low molecular heparin (4000 IU, IV, Q12H) in the hospital and change to rivaroxaban 20 mg daily after discharge. The final treatment included prednisolone 60 mg daily for idiopathic eosinophilia, rivaroxaban 20 mg plus aspirin 100 mg daily for intracardiac thrombus, and digoxin 0.125 mg and diuretics (furosemide 20 mg and aldactone 20 mg) daily for HFpEF. The patient showed substantial clinical improvement and was discharged from the hospital after 1 week of combined treatment. The patient was asked to return to the outpatient department regularly. At the 2-weeks follow-up, she reported good response with diminution of symptoms and decreased eosinophil count in contrast to her past routine blood test (1.03 × 10^9/L↑ vs 2.06 × 10^9/L↑). The dose of prednisolone was reduced from 60 to 40 mg daily at this time. At the fourth week, her routine blood tests showed a normal eosinophil count (0.4 × 10^9/L), and she complained of no symptoms. Digoxin administration was discontinued at this time, while diuretic withdrawal occurred the next month. On subsequent follow-ups, the eventual plan was to taper the prednisolone dose down by 5 mg every week to zero. The thrombus in the cardiac cavity gradually disappeared (11 mm at the beginning, 10 mm after 1 month, 7 mm after 2 months, 4 mm after 3 months, 0 mm after 1 year). After 6 months of treatment with aspirin plus rivaroxaban 20 mg, this patient continued to take rivaroxaban for 1 year.

## Discussion

Studies have reported that approximately 60% of patients with IHES suffer from heart damage (Ogbogu et al., 2007; He et al., 2020). Cardiac involvement secondary to IHES was reported to be the leading cause of death (Ogbogu et al., 2007; He et al., 2020; Salih et al., 2021). Cardiac injury caused by HES occurs in three stages (He et al., 2020). First, eosinophils infiltrate the myocardium and release cationic proteins, leading to myocardial necrosis and microabscesses. Then, platelets activate and form thrombi on the surface of the damaged myocardium. Due to myocardium damage and necrosis, a late fibrotic phase characterized by endomyocardial fibrosis occurs, which is usually associated with cardiac dysfunction. Thus, it was supposed that IHES leads to a highly hypercoagulable state, while a few reports of IHES patients with intracardiac thrombus are being published (Zemleduch et al., 2022). Here, we documented a patient who presented with clinical manifestations of heart failure accompanied by an oblivious intracardiac thrombus, which could help us further understand the etiology and complications of IHES. Besides, this patient was treated with NOACs as her long-term anticoagulant therapy instead of VKA, which is relatively less common.

The patient was initially diagnosed with heart failure, so we needed to determine the etiology of heart failure for this patient. Primary myocardial damage is usually the main cause of heart failure, including ischemic heart disease, cardiomyopathy, myocarditis, myocardial toxicity, metabolic disorders, and immune damage (Nakamura and Sadoshima 2020; Tschope et al., 2021). This patient denied hypertension, diabetes, thyroid disease, drug abuse history or pregnancy. Based on the cTnI elevation, ECG changes, MRI results and clinical manifestations, this patient was considered to have suffered from ACS. However, only mild atherosclerosis was found in the left anterior descending artery. Besides, we have performed pulmonary CTA to rule out PE. Finally, the obviously increased eosinophils in routine blood tests drew our attention and caused us to propose that this patient might suffer from eosinophilic myocarditis, which in turn induced heart failure. Thus, we performed the bone marrow aspirate and bone marrow biopsy. Both results showed marked myelodysplastic activity and a significant increase in eosinophils with no signs of hematologic malignancy. Considering that IHES diagnosis is nonspecific and that discernible secondary causes of eosinophilia need to be excluded, we next conducted a test for inflammatory cytokines (including IL-5), anti-phospholipid antibodies, anticardiolipin, tumor markers and parasites, the results of which were all normal. Due to the highly elevated peripheral eosinophil numbers and increased eosinophils in the bone marrow, as well as the exclusion of secondary causes of hypereosinophilia, the patient was finally diagnosed with IHES and IHES-mediated heart failure.

According to peripheral eosinophil count, hypereosinophilia can be divided into three grades (Costagliola et al., 2020): 1) Mild: The absolute number of eosinophils is less than 1.5 × 10^9^/L (1,500 mm^3^), accounting for less than 15% of leukocyte classification; 2) Moderate: Absolute logarithm of eosinophils is (1.5–5) × 10^9^ (1,500–5,000 mm^3^), accounting for 15%–49% of the classification; 3) Severe: The absolute number of eosinophils is more than 5 × 10^9^/L (5,000 mm^3^), accounting for 50%–90% of the classification. In this case, our patient had moderate hypereosinophilia with an initial eosinophil number of 2.06 × 10^9^/L↑. Thus, we prescribed only oral prednisone 60 mg daily instead of an intravenous injection of corticosteroids.

The aim of the therapy is to reduce the number of eosinophils and to antagonize heart failure and thromboembolic complications. Currently, there are two main theories on how derangement of eosinophil production occurs in HES, which are overproduction of IL-5 and clonal proliferation due to a defect in signal transduction or hematopoietic stem cells (Wang et al., 2021). According to current reports, HES is classified into six main groups (Klion 2015; Rosenberg et al., 2022): 1) myeloproliferative HE/HES (MHES), characterized by FIPIL1-PDGFRA fusion gene rearrangements; 2) lymphocytic variant HE/HES (LHES), characterized by significantly increased IL-5; 3) overlap HE/HES, characterized by involvement of only a single organ system; 4) associated HE/HES, defined as HES in the setting of an underlying cause; 5) familial HE/HES; and 6) idiopathic HE/HES (IHES). The treatment for each subgroup is different. Usually, the etiology for associated HES should be identified, and treatment is targeted at the underlying cause with no direct effect on the eosinophilia. Mepolizumab, an anti-IL-5 antibody, is usually prescribed for MHES patients, while imatinib is the mainstay of treatment for MHES (Kuang and Klion, 2017; Schuster et al., 2020). For other HES therapies, oral corticosteroids remain the first-line therapy, but the availability of hydroxycarbamide and interferon-α are necessary if patients do not have a good response to oral corticosteroid administration alone (Schuster et al., 2020). For our patient, examination of the peripheral blood did not show an increased IL-5 concentration or abnormal expression of FIPIL1-PDGFRA. These results led to the final diagnosis of IHES. High-dose prednisolone 60 mg daily was prescribed. In addition, digoxin and diuretics were used to antagonize heart failure. However, for anticoagulant therapy, there are no guidelines for IHES patients. Current evidence recommends that post-MI patients with intracardiac thrombus should be administered a vitamin K antagonist (VKA) for at least 3 months. However, to maintain a stable international normalized ratio (INR) of 2–3, the use of VKA may be limited by many factors, such as interactions with various foods and drugs, bleeding complications, and a narrow therapeutic window requiring frequent monitoring. In a review of the literature, we found non-VKA oral anticoagulants (NOACs) have been attempted as an off-label use for the treatment of intracardiac thrombosis in case series and reports. In a single-center retrospective study, direct acting oral anticoagulants (including rivaroxaban) therapy appears promising for the treatment of LV thrombus (Fleddermann et al., 2019). Also, researchers pointed out that the NOACs (including rivaroxaban) may be a safe and effective therapeutic option for intracardiac thrombosis after systematically review the related literatures (Ghaffarpasand et al., 2018). Thus, we finally chose rivaroxaban as long-term anticoagulant therapy for this patient. The thrombus decreased in size but could still be detected in the cardiac chamber after taking rivaroxaban 20 mg plus aspirin 100 mg daily for 6 months. Thus, this patient continued to be treated with rivaroxaban 20 mg daily until 1 year later, when the intracardiac thrombus disappeared. This patient is still in follow-up. After 1 year, her physical condition was essentially normal, and she stopped all medication. To date, she has not reported any clinical symptoms.

In conclusion, the occurrence of IHES associated with intracardiac thrombus is rare. From this case, treatment with new oral anticoagulants (NOACs) could be a suitable and effective treatment choice. We will continually follow-up on subsequent prognosis.

## Data Availability

The original contributions presented in the study are included in the article/Supplementary Material, further inquiries can be directed to the corresponding author.
